# Vitamin K and Bone Metabolism: A Review of the Latest Evidence in Preclinical Studies

**DOI:** 10.1155/2018/4629383

**Published:** 2018-06-27

**Authors:** Solmaz Akbari, Amir Alireza Rasouli-Ghahroudi

**Affiliations:** ^1^Periodontics Department, Dental School, Tehran University of Medical Sciences, Tehran, Iran; ^2^Periodontics Department and Dental Implant Research Center, Dental School, Tehran University of Medical Sciences, Tehran, Iran

## Abstract

Bone is a metabolically active tissue that renews itself throughout one's life. Cytokines along with several hormonal, nutritional, and growth factors are involved in tightly regulated bone remodeling. Accordingly, vitamin K as a multifunctional vitamin has been recently deemed appreciable as a topic of research as it plays a pivotal role in maintenance of the bone strength, and it has been proved to have a positive impact on the bone metabolism. Vitamin K exerts its anabolic effect on the bone turnover in different ways such as promoting osteoblast differentiation, upregulating transcription of specific genes in osteoblasts, and activating the bone-associated vitamin k dependent proteins which play critical roles in extracellular bone matrix mineralization. There is also credible evidence to support the effects of vitamin k2 on differentiation of other mesenchymal stem cells into osteoblast. The main objective of the present paper is to comprehensively outline the preclinical studies on the properties of vitamin K and its effects on the bone metabolism. The evidence could shed light on further clinical studies to improve osteogenesis in bone graft surgeries.

## 1. Introduction

Bone is a metabolically active tissue that undergoes constant remodeling throughout one's life. Old bones are regularly resorbed by osteoclasts and constantly replaced with newly formed ones. However, the process of bone remodeling in some parts of the body may reduce the bone mass and result in bone deformation. After tooth extraction, for instance, there is profound resorption often observed in the alveolar crestal bone [1]. This may later interfere with the ideal rehabilitation of the edentulous site with dental implants. Bone adequacy around dental implants has been well documented as a prerequisite for implant osseointegration and its survival in long run [2]. Different bone grafting techniques and bone substitutes have been suggested for bone augmentation [3, 4]. In this regard, various growth factors [5, 6] (such as bone morphogenetic proteins [7]), drugs (such as simvastatin [8]), and nutrients (such as vitamin D [9]) have been evaluated to promote bone formation.

Attempts are ongoing to find materials and techniques to stimulate bone cells and their progenitors to produce native bone at desired sites. Vitamin K is a multifunctional vitamin, which has gained the spotlight for its efficacy for enhancing bone turnover. Vitamin K promotes bone formation by stimulating the osteoblast differentiation, increasing the level of some bone formation markers (e.g., alkaline phosphatase and insulin-like growth factor [10]), and regulating the extracellular matrix mineralization through Y-glutamyl carboxylation [11]. Additionally, vitamin K prevents bone resorption via its anticatabolic activities, namely, decreasing osteoclast differentiation and inhibiting osteoblast apoptosis [10].

This review article aims to comprehensively review the properties of vitamin K and its effect on bone metabolism in preclinical studies. Such evidence may serve as a basis for clinical studies to enhance osteogenesis in bone graft surgeries.

## 2. What Is Vitamin k?

Vitamins are essential to boost physical well-being, and doubtlessly vitamin deficiency can have serious health consequences. Vitamin K is a fat-soluble vitamin first identified in a study on blood coagulation by Carl Peter Henrik in Denmark. The letter “K” stands for “Koagulation”, a Danish term for coagulation. Vitamin K is significantly at play in a wide range of biological activities including regulation of calcium metabolism in tissues, cell growth and proliferation, oxidative stress, inflammatory reactions, and blood coagulation and hemostasis. Vitamin K naturally falls in two types: vitamin K1 and vitamin K2. Although it is fat-soluble, its synthetic analogue, known as menadione or K3, is water-soluble, and it is converted to vitamin K2 in the liver. Vitamin K1, also known as phylloquinone or phytonadione, enjoys an herbal origin and is an inseparable part of human diet. Vitamin K is found in many fruits and vegetables (Kiwifruit, avocado, broccoli, green grapes, and lettuce) as well as oils (canola, soybean, and olive oil). A type of this vitamin, dihydrophylloquinone, that is present in some hydrogenated oils, is less prominent in the body than phylloquinone [12, 13].

Vitamin K2 (menaquinone) refers to a group of chemical compounds with a specific formulation. Such compounds share a naphthoquinone ring and a side chain with variable lengths. The chemical formulation of vitamin K2 is MK_n (MK-2 to MK-14) where “n” is the number of remaining chains of isoprenoid. Although most of these isoprene residues are unsaturated, some forms of menaquinones which are produced by bacteria have saturated prenyl units [14]. Menaquinones, except for MK-4, are synthesized by bacteria. Anaerobic bacteria present in the colon are capable of synthesizing MK-10 to MK-13. Found in fish, liver, milk, vegetables, and eggs, MK-4 is the dominant form of vitamin K in human body where it is primarily produced by conversion of menadione (vitamin k3), directly synthesized from the dietary phylloquinone [15, 16]. As demonstrated by Suhara et al. human osteoblasts could apply the same procedure to produce MK-4 [17]. It has been suggested that the types of vitamin K2 (e.g., Mk-7) with longer chains can be converted to MK-4 as well [18].

Absorbed in small quantities, vitamin K commences the cycle of absorption in the small intestine and it is delivered to the liver and other tissues via the lymphatic system. The major portion of vitamin K1 is stored in the liver and the rest will join the vitamin K2 to be transferred by the low density lipoproteins to other tissues [12]. In human body, MK-4 to MK-10 vitamins are absorbed in greater amounts and show a higher biological activity than K1. Mainly stored in the liver, vitamin K is found in small quantities in body. Liver stores vitamin K1 and long-chain forms of vitamin K2. Brain as well as glands such as pancreas and genital organs are among the other sites storing MK-4 [19]. Therefore, vitamin K deficiency does not equally affect all tissues. For instance, liver, the main reservoir of vitamin K is the last organ to be affected in case of insufficiency or deficiency [20]. There is a body of evidence linking the shortage of vitamin K with increased risk of cancer, cardiovascular disease, soft tissue calcification, and osteoporosis [21–24].

## 3. Vitamin K Cycle

As described below, vitamin K is recycled through a cycle referred to as the vitamin K cycle. This enables the body to recycle and reuse vitamin k as many times as required to obviate the need for dietaries [25]. The cycle operates as follows.

**Figure 1:**
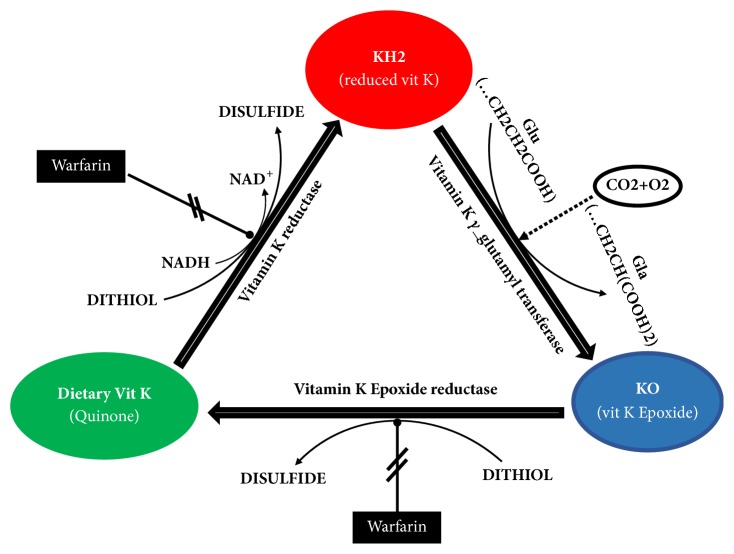
The scheme showing the steps of the vitamin K cycle, the enzymes involved, and the inhibitory action of warfarin.

Vitamin K enters the cells and functions as the cofactor of the endoplasmic reticulum resident *γ*-glutamyl carboxylase (GGCX), which carboxylates any selected glutamate residues on the target proteins and enables these proteins to bind to calcium [11, 26]. Subsequently, vitamin K reductase enzyme converts dietary vitamin K to its reduced form (hydroquinone) which allows GGCX to load a –COOH group on specific proteins. After carboxylation, vitamin K epoxide reductase converts back the resultant oxidized form of vitamin k to hydroquinone (Figure 1).

Worth to be mentioned is the fact that Coumarin (i.e., warfarin), an anticoagulant drug, is the antagonist of vitamin K epoxide reductase and interferes with the vitamin K recycling.

## 4. Biological Activity

Different types of vitamin K vary in their biological activities. This is triggered by the discrepancies in enzyme affinity and tissue distribution. Vitamin K1 is mainly stored in the liver; thus, it plays a greater role in production of coagulation proteins, while vitamin K2 is extensively distributed in the human body [27]. Vitamin K2 enjoys a higher affinity to *γ*-glutamyl carboxylase than that of vitamin K1. Different subtypes of vitamin K2 also differ in the levels of bioactivity and enzyme affinity. For example, MK-7 has the greater bioavailability and plasma half-life than do vitamin K1 and MK-4 [15]. Furthermore, MK-7 overtakes MK-4 and vitamin K1 in the level of anti-NF-*κ*B activity; however, MK-7 cannot induce growth differentiation factor and stanniocalcin genes, identified as the target genes for MK-4 in osteoblasts [28].

## 5. Vitamin K Dependent Proteins

The main function of vitamin K is to serve as a cofactor of GGCX in production of vitamin K dependent proteins (VKDP). To date, 14 VKDPs have been identified, albeit with a shallow account of their functions. Vitamin K dependent proteins include seven proteins (II, IIV, IX, X, protein S, protein X, and protein Z) involved in blood coagulation and synthesized in the liver. There are also four proteins of transmembrane Gla family. Found in bones, the other VKDPs could be listed as Osteocalcin, matrix Gla protein, growth arrest specific 6 protein (Gas 6), and protein S [14, 29, 30].

One of the main noncollagenous proteins found in the bones is Osteocalcin (OC). Also known as bone-Gla-protein, OC is secreted by osteoblasts and some other cells [31]. OC binds to calcium ions and hydroxyapatite crystals. This way, OC seems to be able to exert its regulatory effects on the organization of the bone extracellular matrix and modulates the size and shape of the hydroxyapatite crystals [11, 32]. Transcription and translation of OC gene are regulated by 1,25(OH)_2_ D_3_ [33], but its ability to bind to calcium ions depends on the vitamin K that is responsible for *γ*-carboxylation of three glutamic acid residues in positions 17, 21, and 24 in OC molecule [11].

Evidence supports the role of OC in different physiological activities other than bone metabolism such as glucose metabolism [32], energy metabolism, fertility [34], and ectopic calcification [35]. Based on their degree of carboxylation, various forms of OC differ in their affinity to Ca ions. Since uncarboxylated and undercarboxylated forms of OC demonstrate low affinity to hydroxyapatite, they are easily released into the blood circulation. The circulating OC is used as a good biomarker of bone formation. Irrespective of the concentration of vitamin K, the plasma concentration of OC is correlated to bone turnover and metabolism. However, uncarboxylated OC level is vitamin K dependent [32, 36], and it is considered as an indicator of vitamin K status [37].

Matrix Gla protein (MGP), an extensively studied extra-hepatic Gla proteins, is synthetized by chondrocytes, osteoclasts, and vascular smooth muscle cells. According to the findings in both animal and human studies, MGP inhibits the calcification of arterial media and cartilages, while facilitating normal bone metabolism. It has been demonstrated that MGP attains optimal biological activity just after posttranslational carboxylation [38, 39].

Gla-rich protein and periostin are two other vitamin K dependent proteins, supposed to regulate extracellular matrix mineralization of the bones [32]. Last but not least, a VKDP, protein S, is mainly synthetized in the liver and involved in the anticoagulation pathway. It is also secreted by osteoblasts and involved in the bone turnover in an unclear mechanism [40].

**Figure 2:**
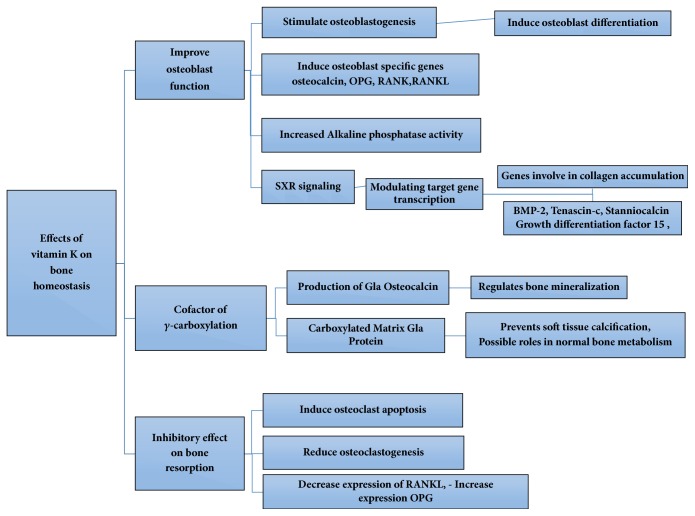
Mechanisms of action of vitamin k on bone homeostasis.

What follows is a more detailed account of the effects of vitamin K on bone cells behaviors. The various effects of vitamin k on bone are also summarized in Figure 2.

## 6. Effects of Vitamin K on Osteoblast Function

Vitamin K affects the proliferation and differentiation of osteoblasts. It prevents the induction of apoptosis in osteoblasts and inhibits Fass-mediated apoptosis in a dose-dependent manner [41, 42], but more effectively improves the osteoblast function [28]. As suggested in the literature, vitamin K2 treatment of osteoblasts could increase both the alkaline phosphatase activity [42–44] and the level of bone anabolic markers such as OC [45, 46] in the cell medium. The more the alkaline phosphatase activity is, the more the formation of the organic matrix and mineral part of the bone is, and so is the deposition of OC and hydroxyapatite in the bone.

Essential role of vitamin K in osteoblastic function through *γ* carboxylation pathway is well established. However, vitamin K2 performs some of its osteoprotective functions by upregulating bone marker genes. Vitamin K2 activates the steroid and xenobiotic receptor (SXR) [28, 47–50] and operates as a transcriptional regulator of the number of osteoblastic biomarker genes and extracellular matrix related genes. The SXR, also known as the pregnane X receptor (PXR), is a nuclear receptor which modulates gene transcription [51] and its protective role in bone metabolism has been shown in PXR-knockout mice study [52]. Research has the fact that vitamin K2 can induce upregulation of CYP3A4 (target gene of SXR) and activation of MSX2 (target gene for PXR) [28]. Menaquinone-7 upregulates Tenascin C and bone morphogenetic protein-2 (BMP-2) genes expression [47]. The effects of vitamin K are not still limited to these pathways. Ichikawa et al. (2007) observed that MK-4, through a pathway independent of SXR and *γ* carboxylation, resulted in activation of two genes, namely, growth differentiation factor 15 and Stanniocalcin. Induction of these genes is exclusive to MK-4, and vitamin K1 and MK-7 do not have such effects. The authors suggested that MK-4 regulates the expression of its target gene through a mechanism dependent on phosphorylation of protein kinase A [53].

Moreover, vitamin K2 supports bone formation and suppresses bone resorption by stimulating the expression of cytokines such as osteoprotegerin (OPG) and inhibiting the expression of receptor activator of nuclear factor kappa-B ligand (RANKL) on osteoblasts/osteoclasts and by this way improves osteoblast differentiation [42, 45]. In the same vein, Yamagushi et al. (2011) observed that vitamin K2 induced downregulating NF-*κ*B (cytokine-induced nuclear factor*κ*) activation in osteoblasts, which is a process independent of *γ* carboxylation mechanism [54].

As illustrated by the results of the studies in vitro, vitamin K (K2 in particular) improves the function of osteoblasts by inducing their proliferation, decreasing their apoptosis, and increasing the expression of osteogenic genes. It also has positive effects on the bone turnover and accordingly regulates bone metabolism.

Effect of vitamin K on osteocyte function also investigated in some in vitro and animal studies. Two osteoporotic rat models demonstrated that vitamin K2 ameliorates adverse effects of glucocorticoid treatment and/or sciatic neurectomy on osteocyte density and lacunar occupancy and had an additive effect on cortical porosity [55, 56]. In a cell culture study Atkins et al. provided the evidence that vitamin K promotes osteoblast transition to osteocyte. They observed that incubation of human osteoblasts with vitamin K2 in a collagen gel medium increased the number of osteocyte-like cells with elongated cytoplasmic processes. This effect seems to be independent to *γ* carboxylation pathway [57]. Vitamin K2 also has regulatory effect on the transcription of bone markers in murine osteocytes [50].

## 7. Effects of Vitamin K on Bone Resorption and Osteoclast Function

Vitamin K2 treatment has been reported to have an inhibitory effect on osteoclastic bone resorption in murine osteogenic culture [58–60], rabbit model [61], and different rat models [44, 62]. Vitamin K prevents bone resorption via several mechanisms. It prevents osteoclast formation either directly or indirectly; that is, it could interfere with the expression of RANKL and upregulates the expression of OPG on osteoclast precursors. In addition, vitamin K decreases both proliferation of tartrate-resistant acid phosphatase positive (TRAP +) cells and TRAP activity in osteogenic culture medium [42, 44, 61]. Moreover, vitamin K2 inhibits bone resorption, induced by bone resorbing factors such as PGE2, IL1*α*, and 1, 25(OH) 2D3 in a dose-dependent manner [42, 58–60].

Yamaguchi et al. (2011) observed that vitamin K2 downregulated basal and cytokine-induced NF-*κ*B activation in human and murine monocytic cell lines and thereupon prevented the bone resorption. Activation of NF-*κ*B signal transduction pathway is essential for osteoclast formation. Hara (1995) stated that the inhibitory effect of menaquinones on bone loss was probably independent of the mechanism of action of *γ* carboxylation and it was triggered by the side chain of vitamin K2 (geranylgeraniol). An interesting finding was that such an inhibitory effect was absent in the side chain of vitamin K1 [59].

Although in vivo [63] and in vitro [61] studies showed the potential of vitamin K2 to induce osteoclast apoptosis, a study on ovariectomized rats revealed that, after MK-4 dietary supplementation (50mg/kg a day), there was a decline in osteoclast bioactivity, yet there was no trace of osteoclast apoptosis [44].

In sum, the current evidence suggests that vitamin K2 reduces osteoclastic activity via different strategies and that it applies an anabolic effect on the bone.

## 8. Effects of Vitamin K on Different Osteoporotic Rat Models

Several animal models have been used to study the effects of vitamin K on the bone metabolism. The studies on ovariectomized rats [44, 62, 64], on unilaterally sciatic neurectomized rats [65, 66], and in tail-suspended rats [67, 68] found that vitamin K treatment had significant positive effects on the bone health. Histologic and microcomputed tomographic evaluations demonstrated that vitamin K2 supplementation inhibited the loss of bone mass density and trabecular bone, improved osteoblast function, and enhanced the serum level of the bone anabolic markers. Accordingly, vitamin K2 improves bone architecture dose-dependently. The highest concentrations used in animal studies have been 30 and 50 mg/kg, which seem to be close to the pharmacologically effective level of this vitamin.

Vitamin K1 and K2 administration elevated serum level of OC (as an indicator of osteoblast function) in high fat diet mice [45]. Osteoblasts and adipocytes differentiate from the same stem cells. Thus, when the number of adipocytes increases as a result of obesity, the number of osteoblasts decreases and causing bone loss [69]. Kim et al. (2013) observed that vitamin K administration in high fat diet mice resulted in a growth in the indices of the bone formation and a reduction in the indices of the bone resorption; however, vitamin K supplementation had no significant effect on the bone metabolism in normal diet mice [45].

Osteoporosis is a systemic skeletal disease and its prevalence increases with age. It often develops asymptomatically and silently and results in reduction of the bone mineral density and bone strength [70]. Literature has addressed the health-enhancing benefits of vitamin K supplementation in osteoporosis [21, 71]. Some animal studies investigated the effect of coadministration of vitamin K2 and other bone acting drugs on osteoporosis. Coadministration of vitamin K2 and Teriparatide (low dose parathyroid hormone derived peptide) improved the osteoblast function and increased the serum level of Gla-OC. These effects were greater in combined use compared to the occasion when vitamin K2 and TPTD were administered in isolation [64].

Bisphosphonates are among the commonly prescribed medications for osteoporosis. They can effectively decrease the risk of bone fracture, hamper the activity of osteoclasts, and decrease the bone turnover; the result is a desirable increase in the bone mineral density. Bisphosphonate-related osteonecrosis of the jaw is among the adverse complications of bisphosphonates, which is hard to treat and its occurrence should be taken into account in planning the dental treatment especially for the patients who take bisphosphonates for long periods of time [72, 73]. Effect of combined use of vitamin K2 and bisphosphonate on osteoporosis was evaluated in rat models. It was recommended that vitamin K2 could ameliorate the suppressive effect of bisphosphonates on bone turnover and increase the bone volume as well as the bone formation parameters [68, 74]. Other reviews emphasized the potentially positive effect of combined treatment with bisphosphonate and vitamin K2 for preventing the fractures in postmenopausal osteoporotic women [75, 76].

Diabetes mellitus is among the most common metabolic disorders worldwide. Diabetes and obesity have been shown to be associated with osteoporosis. Poon et al. (2015) demonstrated that combined use of vitamin K2 and 1,25(OH)_2_D_3_ enhanced calcium deposition and OC expression in osteoblasts of lean and obese diabetic mice; these effects were greater on the obese diabetic group. The results indicated that a single administration of these vitamins could not significantly improve bone anabolic factors such as OC and alkaline phosphatase activity. Thus, combination therapy with vitamin K2 and 1,25(OH)_2_D_3_ was suggested as a promising modality for the treatment of diabetes-associated osteoporosis [46]. Human studies also reported coadministration of vitamin D and K to have beneficial impacts on prevention of bone loss [77, 78].

## 9. MK-7 and Bone Metabolism

MK-4 enjoys the highest bioactivity among different compounds of menaquinones, and it has been the most extensively studied. MK-7, however, has higher bioavailability and a longer half-life than vitamin K1 and MK-4. Natto (fermented soy) is a good source of this vitamin produced by* Bacillus subtilis* [79] and builds a part of the diet in different cultures around the world. Thus, several studies have exclusively evaluated the effects of this vitamin on the activity of osteoblasts and osteoclasts in human and murine cell culture media.

MK-7 affects bone formation by enhancing the function of osteoblasts. This compound results in upregulation of SXR target gene, i.e., CYP3A4 in osteoblasts [28], and induces the synthesis of OPG and OC in osteoblasts. Both of these compounds are osteogenic markers [47, 80]. Further, MK-7 downregulates NF-*κ*B activation in murine and human osteoblasts and osteoclasts. It was suggested that MK-7 exerts this effect independent of *γ*-carboxylation pathway [54].

## 10. Conclusion

There is burden of evidence supporting the osteoprotective effects of vitamin K2 in bone metabolisms. Vitamins K2, especially MK-4, promotes bone formation by stimulating the differentiation of the osteoblast, regulating the mineralization of the extracellular matrix, upregulating the expression of the bone marker genes, and inhibiting the osteoclastogenesis. Based on these anabolic properties of vitamin k, it could be suggested that adding vitamin k as an adjunct to the bone materials may stimulate bone cells and their progenitors to produce native bone with promising results. A recent study has evaluated the behavior of dental pulp stem cells after being exposed to MK-4 in an osteogenic medium. According to the findings, based on ALP activity and extracellular Ca deposition assay, menaquinone 4 can ameliorate differentiation of dental pulp stem cells into osteoblast and may enhance bone regenerative capacity of cell-based bone tissue engineering therapies [81]. Well-designed RCTs are suggested to determine clinical and histological efficacy of vitamin k on the results of bone augmentation surgeries.
